# Giant Ossifying Lipoma of the Hand: A Case Report and Review of Literature

**DOI:** 10.7759/cureus.57760

**Published:** 2024-04-07

**Authors:** Atanas Panev, Teodora Paneva, Bahram Firoozi, Stefan P Petrov, Georgi P Georgiev

**Affiliations:** 1 Department of Orthopedics and Traumatology, University Hospital Queen Giovanna, Medical University of Sofia, Sofia, BGR; 2 Department of Pediatrics, Specialized Hospital for Active Treatment of Children’s Diseases “Prof. Ivan Mitev”, Medical University of Sofia, Sofia, BGR; 3 Department of Orthopedics and Traumatology, University Multiprofile Hospital for Active Treatment (UMHAT) “St. Anna”, Sofia, BGR; 4 Department of Pathology, University Multiprofile Hospital for Active Treatment (UMHAT) “St. Anna”, Sofia, BGR

**Keywords:** diagnosis, treatment, hand, giant, ossifying lipoma

## Abstract

Lipomas are one of the most common benign tumors of the body, characterized by a slow-growing, painless mass that rarely causes symptoms. Bone metaplasia among the mature adipose cells, however, is a rare condition called osteolipoma. In this article, we present a case report of a 61-year-old lady with a giant osteolipoma of the hand. After a surgical extirpation, she showed a fast recovery, and no recurrence during the two-year follow-up period was observed. We aimed to make a literature review of this pathology, discussing the symptoms, diagnosis, and management of this rare condition.

## Introduction

Lipoma is a common benign tumor composed of mature fatty cells that predominantly occurs on the abdomen, shoulder, and upper back. It presents as an asymptomatic, slow-growing round or discoid mass with a soft consistency. Lipomas are benign tumors composed of mature adipose cells [1,2]. In the region of the hand, a giant lipoma is accepted with masses greater than 5 cm [3]. When an osseous or chondrus structure is found among the predominant lipomatous structures,, the tumor is referred to as an osteo or chondrolipoma, respectively. Osteolipoma is a benign tumor consisting of a histological lipoma variant associated with bone metaplasia. It is an extremely rare condition, accounting for less than 1% of all lipomas [4]. The present study aims to present a rare case of giant hand osteolipoma and also make a review of the literature concerning this pathology.

## Case presentation

**Figure 1:**
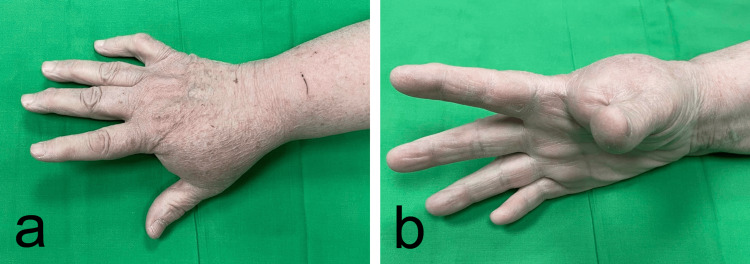
(a and b) Preoperative photography presenting a tumor mass localized in the first dorsal web space

A 61-year-old female patient is admitted to our department with a painful mass on the dorsal side of her right hand. The patient noted the swelling seven years ago, and it had grown slowly thereafter. She had no history of trauma in that area. Clinically, a solid, mobile, regularly-shaped tumor was observed (Figure 1).

**Figure 2:**
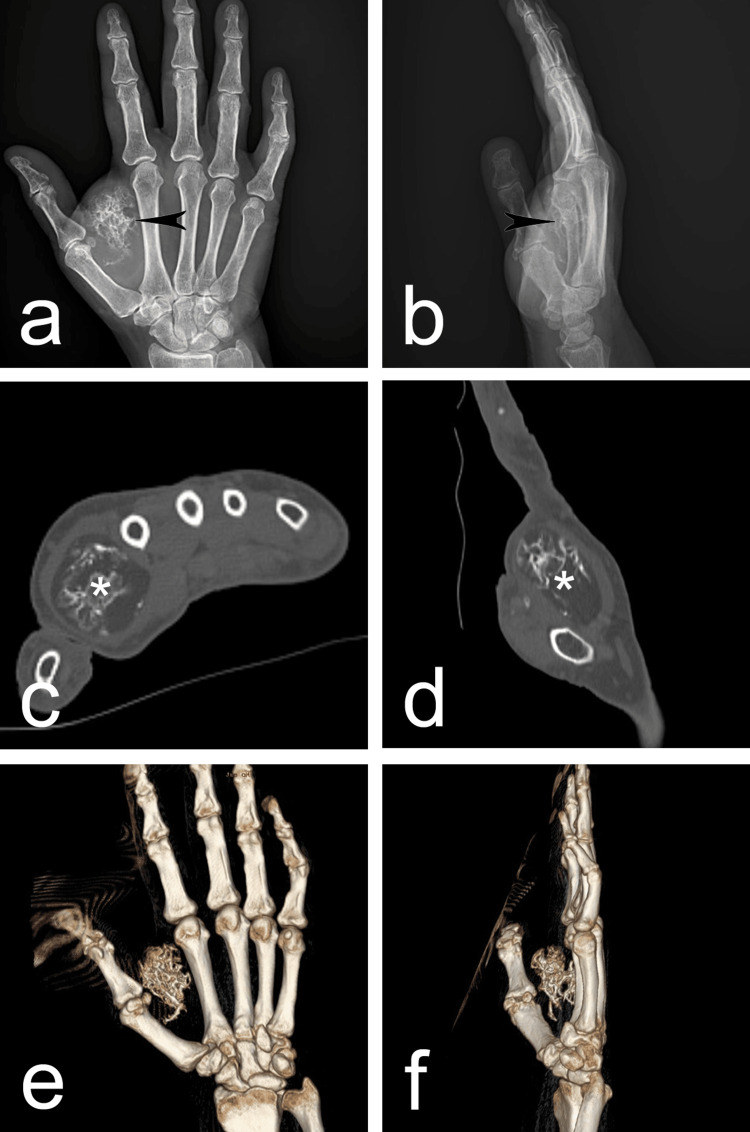
(a-b) Roentgenographies presented a clearly defined radiolucent ovoid formation soft-tissue mass with a trabecular calcified structure in it (arrowhead) in the space between the first and second fingers (a - anterior posterior view; b - lateral view). (c-d) CT imaging presented a soft-tissue mass with netlike calcification in its central part (asterisk) (c - transverse view; d - sagittal view). (e-f) 3D reconstruction CT: computed tomography; 3D: three-dimensional

It was located between the first and second fingers dorsally on the right hand. The range of motion of the thumb was limited. The patient complained of tingling in the first three fingers of the hand. A plain antero-posterior and lateral roentgenographic were performed (Figure 2a-2b). They revealed a soft-tissue mass with a trabecularly calcified structure in the space between the first and second fingers of the right hand (Figure 2c-2d). A CT with 3D reconstruction was performed for further diagnosis. It showed a soft-tissue mass measuring 52/30 mm with netlike calcification in its central part (Figure 2e-2f).

**Figure 3:**
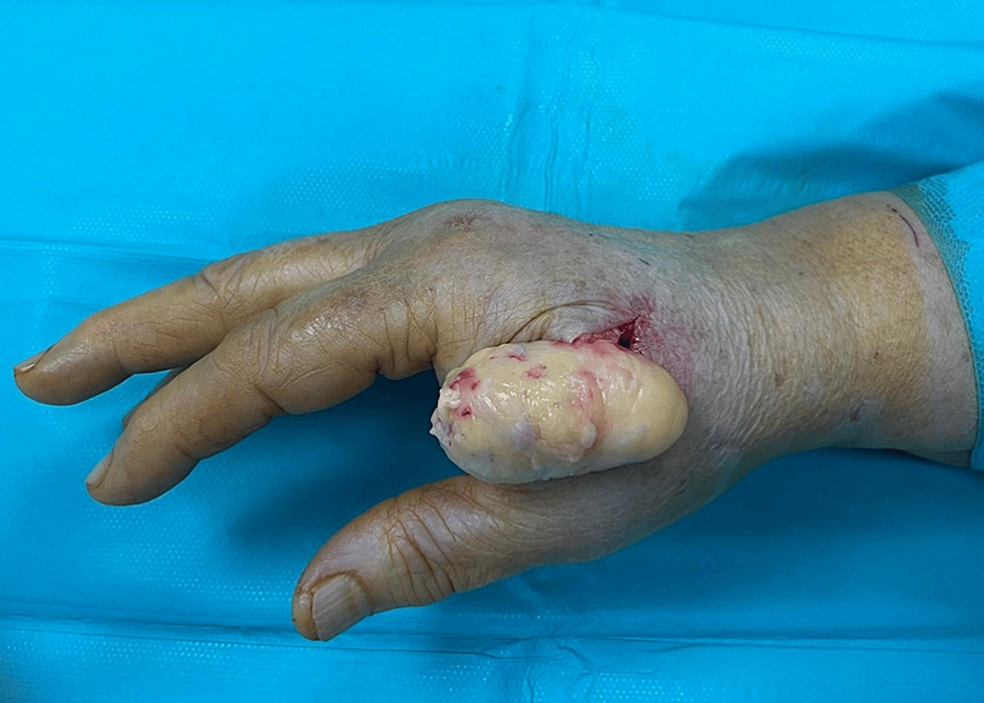
Intraoperative photography presenting a well-encapsulated ovoid mass with macroscopic characteristics of lipoma

The surgery was performed through an arcuate incision over the first web space. Below the skin incision, a tumor with the macroscopic characteristics of a lipoma was observed (Figure 3).

**Figure 4:**
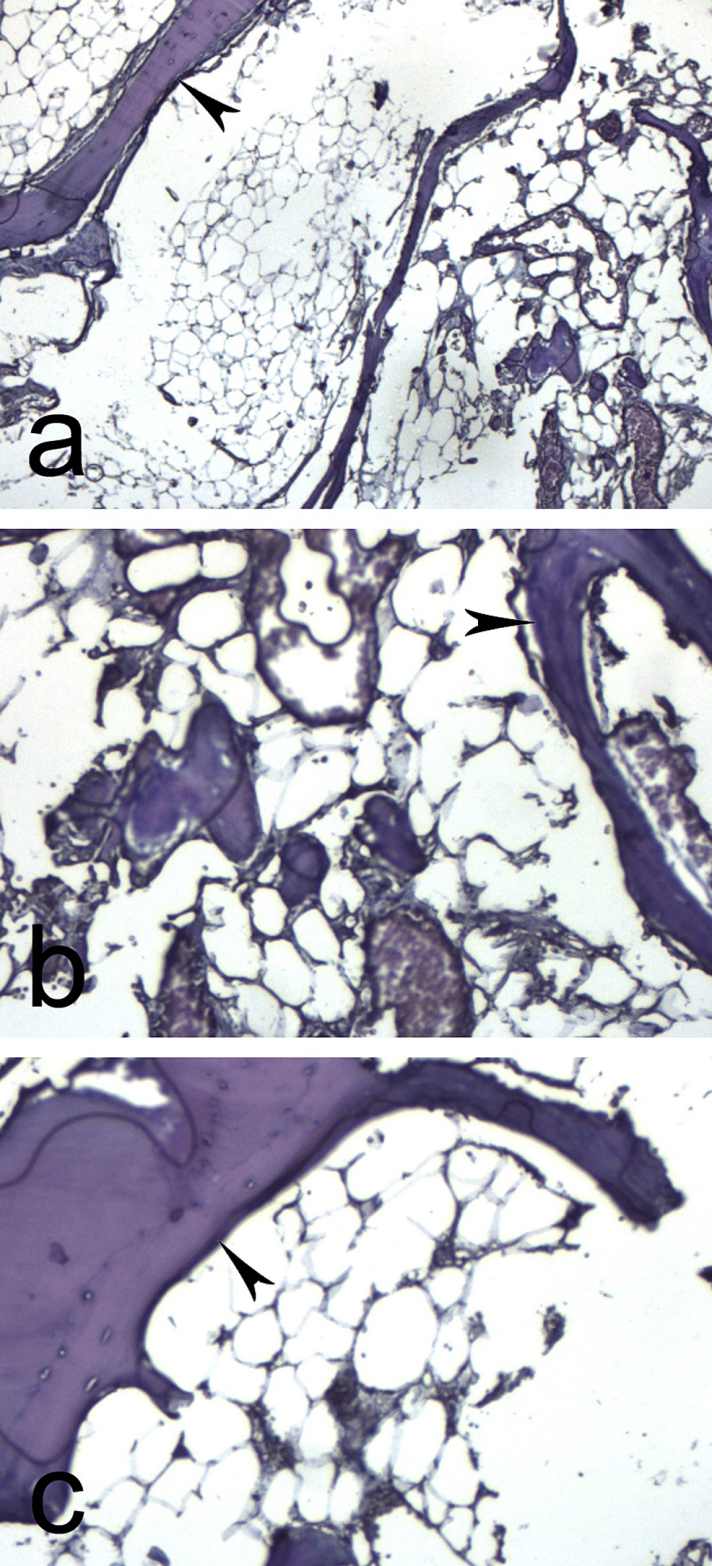
Histopathology of the lesion shows mature adipose tissue with trabecula of mature lamellar bone (arrowhead). (a-b) magnification x 100. (c) magnification x 200

The mass was carefully dissected to ensure complete excision of the tumor; it covered most of the tendon of the flexor pollicis brevis without infiltrating it. Histological investigation presents a diagnosis of osteolipoma (Figure 4a-4c).

Postoperatively, no complications were registered. After two years of follow-up, the patient has a full range of motion of the thumb and no clinical signs of recurrence.

## Discussion

Lipomas with bone structures are extremely rare, described for the first time in 1959 by Plaut et al. [5]. Subsequently, there are a few other descriptions of this tumor, including cases with unexpected locations in the tuber cinereum (in patients with schizophrenia) [6], the external auditory canal [7], and the ankle, elbow, and knee joints [8-10]. Osteolipoma of the hand is rare, with only a few cases reported in the literature (Table 1).

**Table 1:** Presenting cases of osteolipoma of the hand MRI: magnetic resonance imaging

Authors	Age	Symptoms	Duration of symptoms	Size	Diagnostic	Management	Follow-up/recurrence
Tang et al. [11]	8	Congenital osteolipoma	10 months	2.5 cm	X-ray	Surgery	8 years/yes, 19 months, 8 years
Hopkins and Rayan [12]	61	Enlarging mass	6 months	unknown	X-ray, MRI	Surgery	Unknow/no
Teoh et al. [13]	40	Slow-growing mass, painful	3 years	6 x 4 cm	X-ray	Surgery	1 year/no
Chen et al. [14]	54	Enlarging mass	10 years	3.6 x 5 x 3.6 cm	X-ray, MRI	Surgery	1 year/no
van Zwieten and van Unen [15]	41	Slow-growing mass, finger numbness	1 year	2 cm	X-ray, MRI, ultrasound	Surgery	6 months/no
Echavarria et al. [16]	51	Slow-growing mass, pain, finger paresthesia	10 years	6 x 5 x 4 cm	X-ray, MRI	Surgery	6 months/no

The pathogenesis of lipoma with osseous elements is still unclear, and multiple theories exist, although two proposed mechanisms are widely cited in the literature [11,17]. The first theory suggests that osteolipoma results from the multidirectional differentiation of multipotent mesenchymal cells. Tang et al. [11] described a case of congenital osteolipoma. After a careful pathological examination of this tumor, it was described as a benign mesenchymoma and illustrates the pluripotentiality of the mesenchymal tissue. On the other hand, osteolipoma may arise after repetitive trauma, metabolic changes, or probably ischemia, leading to metaplasia of pre-existing fibrous elements within the lipoma and osteoblasts development [17]. The exact mechanism is still unknown, and more examination of the genetic changes might reveal the exact etiology.

The preoperative diagnosis remains challenging. The most common and first-line method of investigation when a hand tumor is suspected is roentgenography. Typically, osteolipoma would appear as a well-defined mass with signs of trabeculae calcification within it. No cortical abnormality should be observed [4]. CT and MRI are excellent modalities for more precise diagnosis. These methods provide better soft-tissue contrast and can identify fatty, ossified, or calcified components [14]. In our case, we performed a CT scan with 3D reconstruction, showing a soft-tissue mass with net-like calcification.

Histologically, osteolipoma consists of mature adipose tissue with a multifocal area of bony tissue (lamellar bone, woven bone, and cancellous bone) [4]. No cellular atypia or increased mitotic index is usually observed [14], which proves the benign nature of the tumor. No nuclear atypia, cellular pleomorphism, mitosis, or necrosis were ever reported in any of the cases we reviewed.

This pathology can cause different symptoms, from a slow-growing painless mass to finger paresthesia and local pain when compressing peripheral nerves [1-9]. In our case, the patient suffered from swelling and a limited range of motion of the thumb.

In terms of differential diagnosis, osteolipoma can be mistaken for parosteal lipomas. Radiologically, in the last one, there is hyperostosis or pathological changes in the surrounding bones that are not observed in the cases of osteolipoma [18]. On the other hand, it can be distinguished from tumor calcinosis, which has a denser pattern of calcification, and myositis ossificans, in which a typical diffuse ossification is witnessed and is always associated with a history of trauma [12].

The treatment of giant lipomas usually consists of complete surgical removal. Due to the fact that these tumors are well-encapsulated, the excision is relatively easy. Intralesional excision through liposuction could also be performed, but with a higher risk for recurrence and neural or vascular injury [1-3].

## Conclusions

The current article aims to present a case report of a giant osteolipoma of the hand. The shown literature review highlights the rarity of this pathology and presents the diagnostic features and treatment. Although it is a benign tumor, osteolipoma can be mistaken for more severe conditions with a bad prognosis. A careful pathohistological and radiological observation must be done.

## References

[1] Slavchev SA, Georgiev G, Penkov M, Landzhov B (2012). Giant lipoma extending between the heads of biceps brachii muscle and the deltoid muscle: case report. J Curr Surg.

[2] Slavchev SA, Georgiev GP (2017). A giant deep-seated lipoma in a child’s forearm. J Hand Surg Asian Pac Vol.

[3] Georgiev G, Telang M, B F, Ananiev J, Landzhov B, Olewnik LH, L S (2022). Giant lipoma of the hand. Acta Med Bulg.

[4] Wong BL, Hogan C (2022). Osteolipoma of head and neck - a review. Braz J Otorhinolaryngol.

[5] Plaut GS, Salm R, Truscott DE (1959). Three cases of ossifying lipoma. J Pathol Bacteriol.

[6] Maravi P, Kaushal L, Uikey A, Sarker M, Sachdeva K (2024). Osteolipoma of tubercinerium: An incidental finding in a patient with schizophrenia. Radiol Case Rep.

[7] Ahmed S, Kunnumal A, Afreen SS, Hasan Z (2023). Parosteal osteolipoma in the external auditory canal: a rare variant of lipoma. Indian J Otolaryngol Head Neck Surg.

[8] Kim S, Ha C, Kwon AY, Choi W (2022). Lipoma with osteocartilaginous metaplasia in infrapatellar fat pad: a case report and review of literature. Medicine (Baltimore).

[9] Zaizi A, El Ktaibi A, Rabah A, Bouabid AS, Boussouga M (2022). Osteolipoma of the ankle: a rare case report. Foot (Edinb).

[10] Jlidi M, Bouaicha W, Ayachi M (2023). Intra-articular osteolipoma of the elbow: a case report and a review of the literature. Bone Rep.

[11] Tang TT, Chamoy L, Meyers A, Babbitt DP, McCreadie SR (1981). Congenital lipoma with ossification in the hand of a child. J Pediatr Surg.

[12] Hopkins JD, Rayan GM (1999). Osteolipoma of the hand: a case report. J Okla State Med Assoc.

[13] Teoh LC, Chan LK, Lai SH (2001). Ossifying lipoma of the hand: a case report. Ann Acad Med Singap.

[14] Chen H-Y, Jim Y-F, Chuang H-Y, Shen W-C (2008). Ossifying lipoma of the hand: a case report with imaging findings. Mid-Taiwan J Med.

[15] van Zwieten G, van Unen JM (2014). A man with a swelling in the palm of his hand (Article in Dutch). Ned Tijdschr Geneeskd.

[16] Echavarría ME, García LJ, Silveira MV (2021). Giant hand osteolipoma: case report. Rev Iberoam Cir Mano.

[17] Demiralp B, Alderete JF, Kose O, Ozcan A, Cicek I, Basbozkurt M (2009). Osteolipoma independent of bone tissue: a case report. Cases J.

[18] Miller MD, Ragsdale BD, Sweet DE (1992). Parosteal lipomas: a new perspective. Pathology.

